# Quorum Sensing versus Quenching Bacterial Isolates Obtained from MBR Plants Treating Leachates from Municipal Solid Waste

**DOI:** 10.3390/ijerph15051019

**Published:** 2018-05-18

**Authors:** Albert Soler, Lucía Arregui, Miguel Arroyo, José Antonio Mendoza, Andrea Muras, Cristina Álvarez, Cristina García-Vera, Domingo Marquina, Antonio Santos, Susana Serrano

**Affiliations:** 1 Department of Genetics, Physiology and Microbiology, Complutense University of Madrid, 28040 Madrid, Spain; albert.sol.her@gmail.com (Al.S.); dommarq@ucm.es (D.M.); ansantos@ucm.es (An.S.); suserra@ucm.es (S.S.); 2 Department of Biochemistry and Molecular Biology I, Complutense University of Madrid, 28040 Madrid, Spain; arroyo@ucm.es; 3 Department of Chemical and Nuclear Engineering, Polytechnic University of Valencia, 46022 Valencia, Spain; jamendoz@iqn.upv.es; 4 Department of Microbiology and Parasitology—CIBUS, Universidade de Santiago de Compostela, 15782 Santiago de Compostela, Spain; andrea.muras@usc.es; 5 URBASER S.A., Camino de Hormigueras, 171, 28031 Madrid, Spain; calvarezr@urbaser.com (C.Á.); cgvera@urbaser.com (C.G.-V.)

**Keywords:** quorum sensing, quorum quenching, AHL, biofouling, MBR, wastewater treatment

## Abstract

Quorum sensing (QS) is a mechanism dependent on bacterial density. This coordinated process is mediated by the synthesis and the secretion of signal molecules, called autoinducers (AIs). *N*-acyl-homoserine lactones (AHLs) are the most common AIs that are used by Gram-negative bacteria and are involved in biofilm formation. Quorum Quenching (QQ) is the interference of QS by producing hydrolyzing enzymes, among other strategies. The main objective of the present study was to identify QS and QQ strains from MBR wastewater treatment plants. A total of 99 strains were isolated from two Spanish plants that were intended to treat leachate from municipal solid waste. Five AHL producers were detected using AHL biosensor strains (*Chromobacterium violaceum* CV026 and *Agrobacterium tumefaciens* NT1). Fifteen strains of seventy-one Gram-positive were capable of eliminating or reducing at least one AHL activity. The analysis of 16S rRNA gene sequence showed the importance of the *Pseudomonas* genus in the production of biofilms and the relevance of the genus *Bacillus* in the disruption of the QS mechanism, in which the potential activity of lactonase or acylase enzymes was investigated with the aim to contribute to solve biofouling problems and to increase the useful lifespan of membranes.

## 1. Introduction

Microorganisms have developed complex communication systems to produce, and, in turn, respond to extracellular signals that are secreted by others [1,2,3]. This microbial communication system, called Quorum Sensing (QS), is the ability to sense their population density through the synthesis and secretion of small molecules, called AutoInducers (AIs) [4,5,6]. Although different types of signal molecules have been described [7], the best characterized QS signals are the *N*-acyl homoserine lactones (AHLs). These QS signals are molecules of low molecular mass that are characterized by having a conserved lactone moiety and a variable acyl side chain (between 4 and 18 carbons) [8]. Despite of AHLs are QS signals that are considered to be typical of Gram-negative bacteria; they are also produced by the Gram-positive strain *Exiguobacterium* MPO [9]. QS controls, via gene expression modulation, a wide range of activities, such as pathogenesis [10,11], bioluminescence, biosynthesis of antibiotics, mobility, or biofilm formation [12,13].

Nowadays, membrane bioreactors (MBR) are becoming an advanced feasible treatment system for both industrial and urban wastewater treatment plants (WWTP) [14,15,16]. This system is a combination of a biological degradation process, primarily an activated sludge process, along with a solid-liquid separation by micro- or ultrafiltration membranes of pore size ranging from 0.05 to 0.4 µm [17]. MBR systems allow for working with high concentrations of mixed liquor suspended solids, and also obtaining low sludge production and excellent effluent qualities necessary for agricultural or domestic uses or to simply incorporate the treated wastewater back into the water cycle with increased guarantees. Despite the advantages described, there are several problems that are hindering the implementation of such systems. Firstly, it has been found that the high economical cost of the membranes make them difficult to use in large scale projects [18]. Secondly, membrane fouling, of chemical, biological, and physical origin is considered to be the biggest obstacle to the implementation of industrial-scale MBR systems at a higher extent [19].

The problem of membrane biofouling that is produced by Gram-negative bacteria could be considered to be related with QS, since it has been identified as one of the mechanisms controlling bacterial biofilm development and play a key role in the formation and maturation of numerous bacterial species biofilms [20,21]. Biofilm formation occurs by the accumulation of different microorganisms that are surrounded by an extracellular matrix consisting of secreted proteins, nucleic acids, polysaccharides, and dead cells. It generates a progressive loss in the efficiency of the membranes [22].

An effective technique for membrane biofouling control, which is based on the enzymatic degradation of signal molecules (AHLs) or Quorum Quenching (QQ), has been proposed [21,23,24,25,26]. QQ is a mechanism that can interfere with any QS-regulated phenotype [27]. At present, four types of enzymes that can degrade AHLs are known [6]: decarboxylases, deaminases, lactonases, and acylases. However, acylases and lactonases seem to be the most promising QQ activities for biotechnological and medical applications [28]. Acylases, which hydrolyze the amide bond generating a free fatty acid and the homoserine lactone ring [27,29,30], have been identified in *Streptomyces* sp. [31], *Pseudomonas aeruginosa* [32], or *Bacillus cereus* [33], among others. Lactonases hydrolyze the core lactone ring of AHL signaling molecules and they have been identified in *Acinetobacter* sp. [34], *Rhodococcus* sp. [35,36], or *Agrobacterium tumefaciens* [37]. Up to now, numerous lactonases have been described in genus *Bacillus* sp. [38,39,40,41,42,43]. All of them belong to most abundant cluster among the AHL-degrading enzymes, the metallo-beta-lactamase (MBL) superfamily. However, several unrelated families with AHL-lactonase activity belong to the phosphotriesterase (PTE) family or the α/β-hydrolase-fold family have been described [28].

Understanding microbial population development in MBR treating leachates from municipal solid wastes is challenging due to the complexity of waste materials that are deposited and the temporal heterogeneity. In the present study, QS and QQ bacteria were isolated from samples of two MBR plants with biofouling problems that are located in Spain with the aim of increasing the life expectancy of the ultrafiltration membranes. The biological control of this problem, through the use of native bacterial isolates with QQ activity, has been proposed as a potential mechanism that could prevent biofouling on membranes of MBR systems, reducing environmental damages, and minimizing operational costs [44].

## 2. Materials and Methods

### 2.1. Wastewater Treatment Plants

Two full-scale MBRs (WWTP-A and WWTP-B) that treat lixiviates from plants recycling the previously separated organic fraction from the municipal solid wastes [45,46] were sampled. In the WWTP-A, the Anaerobic Digestion (AD) is carried out by means of a high solids system (dry process, i.e., solids concentration higher than 15%) and in the WWTP-B, the AD is carried out by means of a low solids system (wet-process, i.e., solids concentration that is lower than 10%). Both WWTPs were designed to eliminate organic matter and nitrogen [46].

For both plants, the MBR configuration was the same, i.e., membranes were external and mixed liquor was pumped from the biological reactor to the UF module. The membranes used in the plants were multichannel tubular and the installed active surface was 127 m^2^ (WWTP-A) and 72 m^2^ (WWTP-B). Biological reactors consisted of one anoxic tank, two aerobic tanks, and a final tank that can be operated aerobically or anoxically, depending on the nitrogen removal efficiencies [46].

### 2.2. Sampling and Strain Isolation

Five samples were collected, between September 2014 and July 2015, from the final tank of the biological reactor of both WWTPs and were maintained at 4 °C until they were processed. For bacterial isolation, aliquots of 1 mL were taken from the samples, and 10-fold dilutions were made serially to a final dilution of 10^−5^. After that, 100 µL aliquots were plated onto four different mediums: Luria-Bertani medium (LB); Reasoner’s 2A Agar (R2A); Violet Red Bile Glucose Agar (VRBG) for the detection and count of the enterobacteria and *Pseudomonas* Selective Isolation Agar (PSIA). Two plates of each dilution were incubated at 28 °C for 1–2 days (LB and R2A medium); for PSIA and VRBG medium, two plates of each dilution were incubated at 37 °C for 4–5 days. For the estimation of CFUs, the plates with 30–300 colonies were selected. For strain isolation, different macroscopic characteristics, such as colony morphology, color, or roughness were used. After several rounds of isolation, pure cultures were subjected to Gram staining. The obtained isolates were maintained on LB medium at 4 °C.

### 2.3. Identification of Isolates

Bacterial isolates were identified by the sequence analysis of the 16S rRNA gene. Genomic DNA from the different isolates was extracted (UltraClean^®^ Microbial DNA Isolation Kit—MO BIO Laboratories, Inc., Carlsbad, CA, USA) and bacterial 16S rRNA gene was amplified using forward Y1 primer (5′-TGG CTC AGA ACG AAC GCT GGC GGC-3′) and reverse Y2 primer (5′-CCC ACT GCT GCC TCC CGT AGG AGT-3′) to amplify a 348-bp fragment from the 16S rRNA gene [47]. PCRs were conducted in a 50 µL reaction volume in thin-walled 0.2 mL tubes. Briefly, 16 µL of DNA template were added to a 25 µL master reaction mixture (Ready-Mix™ Taq PCR Reaction Mix, Sigma-Aldrich, Madrid, Spain), 2 µL of Y1 (50 µM) and 2 µL of Y2 (50 µM) primers, and 5 µL of sterile distilled water. PCRs were carried out under the following standard conditions: initial denaturation at 95 °C for 10 min, followed by 30 cycles that were consisting of denaturation at 95 °C for 45 s, annealing at 58 °C for 1 min, and extension at 72 °C for 45 s, followed by a final extension at 72 °C for 10 min. The PCR-amplified fragments were analyzed by electrophoresis in 1.2% of agarose in TAE buffer and staining with GelRed^®^ (Biotium, Fremont, CA, USA) The amplification efficiency was analyzed under UV light, and images were taken with a digital camera. The amplified fragments were purified with the UltraClean™ PCR Clean-Up PCR purification kit (MO BIO Laboratories, Inc., Carlsbad, CA, USA) according to the manufacturer’s protocol. A total of twenty-one 16S rRNA gene sequences were identified and were compared by BLAST-search (GenBank; http://www.ncbi.nlm.nih.gov). 16S rRNA gene sequences were aligned using CLUSTAL W2 [48]. The sequences were deposited in GenBank under the accession numbers: KU897070 to KU897089 and KU597170 (*Rhodococcus ruber* 54B).

### 2.4. Detection of AHL Production by Agar Plate Assay

A screening was done to detect AHL production by Gram-negative isolated strains. For this assay, two reporter strains were used: *Chromobacterium violaceum* CV026 [49,50] and *Agrobacterium tumefaciens* NT1 [51].

The strains were streaked in parallel to the biosensor strains on LB agar plates for *C. violaceum* CV026, and LB agar plates with 60 µg/mL of X-gal (5-bromo-4-chloro-3-indolyl-β-d-galactopyranoside) for *A. tumefaciens* NT1. The most active agonist AHL for *C. violaceum* CV026 is C6-AHL; other AHLs that induce the production of violacein include C6-3-oxo-AHL and C8-AHL, C8-3-oxo-AHL and C4-AHL reasonably well. *A. tumefaciens* NT1 can produce a blue color from the hydrolysis of X-Gal by the β-galactosidase activity in response to 3-oxo-substituted AHL-derivatives with acyl chain lengths from 4 to 12 carbons and also 3-unsubstituted AHLs, with the exception of C4-AHL. This sensor can also detect 3-hydroxy derivatives, more precisely, C6-3-hydroxy-AHL, C8-3-hydroxy-AHL, and C10-3-hydroxy-AHL [52]. Plates were incubated at 30 °C for 48 h.

### 2.5. Surface Adherence Capability of AHL Producers

Gram-negative strains were cultured in liquid LB medium overnight at 28 °C (120 rpm) and were then diluted to reach an OD600nm of 0.1 in the same medium. Subsequently, 100 µL of each diluted culture were pipetted into each one of eight wells of a sterile non-treated polyethylene 96-well microtiter plate (Corning^®^ Costar^®^ culture plates, Sigma-Aldrich). Sterile LB medium was used as a negative control. Microtiter plates were prepared in duplicate and incubated at 28 °C (120 rpm, 24 h). The adherence capabilities were quantified according to the crystal violet method that was described by Merritt et al. (2005) [53]. Absorbance was measured at 590 nm at room temperature using an iEMS Reader MF (Labsystems, Helsinki, Finland). This assay was carried out in duplicate and eight readings were recorded for each strain. According to Stepanovic et al. (2000) [54], the strains were classified by their adherence capabilities in: (1) non-adherent (Abs < Abscontrol); (2) weakly adherent (Abscontrol < Abs < 2 × Abscontrol); (3) moderately adherent (2 × Abscontrol < Abs < 4 × Abscontrol); and, (4) strongly adherent (Abs > 4 × Abscontrol).

### 2.6. Detection of Quorum Quenching Activity by the Agar Plate Assay

Three different AHL (Sigma-Aldrich, Spain) were used: C6-HSL (*N*-hexanoyl-dl-homoserine lactone), C8-HSL (*N*-octanoyl-dl-homoserine lactone), and C12-HSL (*N*-dodecanoyl-dl-homoserine lactone). To detect QQ activity of the isolated Gram-positive bacteria, both reporter strains mentioned previously and *Aeromonas hydrophila* (strain E2MB52) as negative control, were grown in 4 mL of LB broth at 28 °C and 120 rpm. After 24 h, the strains were diluted to an OD600 nm of 0.5 in the same medium. Subsequently, 5 µL of each AHL stock solution were added to Gram-positive cultures to achieve a final concentration of 0.5 µg mL^−1^, and were incubated at 28 °C and 120 rpm. After 24 h, 50 µL of the supernatants were spotted in duplicate in wells that were made on LB agar plates overlaid with 5 mL of overnight cultures of *C. violaceum* CV026 (or *A. tumefaciens* NT1) in soft LB (0.8% agar). Fifty µL of *A. hydrophila* supernatant and 50 µL of sterile LB that was supplemented with C6-HSL, C8-HSL, or C12-HSL were used as negative controls in all of the plates. Fifty µL of sterile LB were used as positive control.

### 2.7. QQ Strains Addition to Mixed Liquors and Monospecific Bacterial Biofilms

To perform these tests, strains with QQ activity were added to mixed liquors of WWTP-A and WWTP-B (ML_A_ and ML_B_—both diluted to 10^−1^) and *P. aeruginosa* (QS1) cultures. These assays were performed using both, cell cultures and culture supernatants. All of the strains were grown overnight at 28 °C and 120 rpm in sterile tubes containing liquid LB medium and then diluted to reach an OD600nm of 0.1 in the same medium. Subsequently, 50 µL of QQ strains and 50 µL of AHL producers were pipetted into each one of eight wells of a sterile non-treated polyethylene 96-well microtiter plate (Corning^®^ Costar^®^ culture plates, Sigma-Aldrich). One hundred µL of sterile liquid medium were used as a negative control and 100 µL of the AHL producer were used as a positive control. Then, microtiter plates were prepared in duplicate and were incubated at 28 °C and 120 rpm during 24 h and adherence capabilities were quantified using the crystal violet procedure that is cited above.

### 2.8. Effect of QQ Strains on Ultrafiltration Fluxes

Ultrafiltration (UF) tests were performed in an automated UF laboratory plant (Figure S1) that was equipped with elements for the regulation of the cross-flow velocity (CFV), transmembrane pressure (TMP), and temperature. The fouling tests were carried out at the following operation conditions: 1 bar of TMP, 25 °C, and 2 m s^−1^ of CFV. The membrane that was used in every test was a 150 kDa cut-off hydrophilic polyethersulfone membrane (Nadir UH150, Microdyn Nadir, Germany). New membranes were used for each experiment and its initial permeability was measured using deionized water before each UF test with mixed liquor. The duration of each test was 3 h, time required to reach the steady state. Permeate and retentate were recirculated into the feed tank. The permeate flux was monitored with an electronic weighing scale (KERN KB 2400-2N, 0.01 g accuracy, Kern & Sohn GmbH, Balingen, Germany) and the collected data were recorded, every 15 s, with data acquisition software (Balance Connection SCD-4.0, Kern^®^, Kern & Sohn GmbH, Balingen, Germany).

Two types of tests were performed. First, an assay that was based on a direct inoculation of strains with positive QQ, which had been selected as best candidates in the previous experiments, and, second, an assay that is based on the encapsulation with alginate of the QQ strains [24] to achieve the immobilization of cells while maintaining the desired catalytic activity and cellular viability. The total volume for each assay was approximately 3.25 L:1 L of mixed liquor from each WWTP, 2 L of purified water, and 250 mL of bacterial culture in stationary phase (applied directly or entrapped in alginate spheres). For bacteria entrapment exponentially growing bacterial cells were suspended in 2% sodium alginate (*v/v*). This bacterial-alginate mixture was dripped from a height of 10 cm into 100 mL of crosslinking solution (0.05 M of CaCl_2_). The gel formation was achieved with a syringe at room temperature as soon as the sodium alginate drops come in direct contact with the calcium solution. The beads should be fully formed in 30 min. Relatively small (3–4 mm) alginate beads were preferred to minimize the mass transfer resistance.

### 2.9. Quantification of Enzymatic Activities

AHL degradation by lactonases was determined using high-pressure liquid chromatography-mass spectrometry (HPLC-MS). One mL sample, obtained from a 24 h 15 mL LB culture of the selected strains, was centrifuged at 10,000 rpm for 10 min to separate the cells from the culture media. Supernatants were removed and pellets were resuspended with 1 mL of PBS pH 6.5, mixed with 40 µL of C6 or C12-HSL (2 µg mL^−1^), and incubated for 24 h at 22 °C and 150 rpm rotary shaking. 500 µL of the mixture were directly extracted twice with an equal volume of ethyl acetate, evaporated under nitrogen flux at 50 °C, and resuspended in 200 µL acetonitrile to quantify the remaining AHLs. The remaining 500 µL of the mixture were acidified with HCl 5M to pH 2.0 and incubated for 24 h at 22 °C before extraction in order to facilitate the recovery of the AHL activity derived from the hydrolysis of the lactone ring derived from the action of lactonases. Each AHL dissolved in PBS at the same concentration was used as control [55,56]. HPLC-MS analyses were performed with a HPLC 1100 series (Agilent), equipped with a C8 precolumn 2.1 × 12.5 mm (5 µm particle size) and a Zorbax Eclipse XDB-C18 150 × 4.6 mm column (5 µm particle size) [55]. The mobile phase was composed of 0.1% formic acid in water (A) and 0.1% formic acid in acetonitrile (B) [57]. The elution conditions were described by Romero et al. (2011) [55]. MS experiments were conducted on an API4000 triple-quadrupole linear ion trap mass spectrometer (Applied Biosystem, Foster City, CA, USA) equipped with a TurboIon source using positive ion electrospray, multiple reaction monitoring (MRM) mode [55,56,58]. The MRM signals were used to generate relative quantification information by comparison with a calibration curve that was constructed for molecular ion abundance, using each of the appropriate AHL synthetic standards [59].

PVA activity was assayed by the PDAB method, which is based on the formation of a Schiff base when 6-aminopenicillanic acid has reacted with *p*-dimethylamino benzaldehyde (PDAB) [60]. Cultures were grown in 50 mL Falcon^®^ flasks (Thermo Fisher Scientific, Majadahonda, Madrid) containing 15 mL of LB medium (24 h at 28 °C and 120 rpm). After centrifugation (9000× *g*), the cells were removed. The proteins of the supernatant were precipitated with 70% (*v/v*) ethanol, and, after centrifugation at 4000× *g* for 30 min, the precipitate was resuspended in 135 µL 1 M phosphate buffer (pH 8). Then, the PVA acylase activity was determined at 40 °C for 60 min. Reaction mixture contained 150 µL of penicillin V (45 mg mL^−1^), 135 µL of resuspended supernatant, and 15 µL of 1 M potassium phosphate buffer (pH 8). To stop the reaction, 900 µL of 20% acetic acid water solution were added in an ice bath. Samples were centrifuged (2 min, 9000× *g*) and 250 µL of the supernatant transferred to 100 µL of PDAB (0.5% *w/v* in methanol). Then, the absorbance at 405 nm was recorded for the determination of 6-APA concentration (Balasingham et al., 1972). One unit of enzyme activity was defined as the amount of enzyme that is required to produce 1 µmol 6-APA/min at pH 8 and 40 °C [61].

## 3. Results

### 3.1. Identification and Characterization of Bacterial Isolates

A total of 99 bacterial strains (48 strains from WWTP-A and 51 from WWTP-B) were isolated from MBR activated sludge, with cellular densities that were ranging from 4.5 × 10^5^ CFU mL^−1^ to 2.95 × 10^7^ CFU mL^−1^, using culture-dependent techniques on LB, R2A, VRBG, and PSIA media. Seventy-one strains were Gram-positive and the other 28 strains were Gram-negative. Comparison of the nearly complete 16S rRNA nucleotide sequences confirmed that the isolates belong to four different genera *Bacillus*, *Gordonia*, *Pseudomonas,* and *Aeromonas*, with a predominance of *Bacillus cereus* and *Pseudomonas aeruginosa* in all cases, similarity values among the isolated strains and the closest relative strains were between the 99% and the 100% (Table S1). A *Rhodococcus* strain from our laboratory collection was also included in the research (*Rhodococcus ruber* 54B—accession number KU597170) due to the reported ability of *Rhodococcus* to prevent biofouling in MBR [23,24,26,62].

### 3.2. AHL Producers and Potential Adherence to Surfaces

Twenty-eight Gram-negative strains were tested against the reporter strains (*C. violaceum* CV026 and *A. tumefaciens* NT1). *C. violaceum* CV026 responded positively only to *A. hydrophila* E2MB52, indicating the production by this strain of short or medium-chain AHLs. *A. tumefaciens* NT1 responded to five bacterial isolates (*A. hydrophila* E2MB52; *P. pseudoalcaligenes* E2MB82; *Thauera butanivorans* E2DN83; *P. aeruginosa* E2MBUPVDN12; and, E2MBUPVDN20), suggesting that those strains produce medium or long-chain AHLs.

The five AHL producers, the 5.1% of the total isolates (17.8% of the Gram negatives), were used to test their adherence and subsequent ability to form biofilms on polyethylene surfaces. All of them reached OD 590 nm above the average value of the control (sterile medium) at 24 h of incubation, although the strain E2MB82, which is a *P. pseudoalcaligenes*, clearly recorded the highest absorbance values (Figure S2), indicating high effectiveness in surface colonization (Table 1).

### 3.3. Inhibition of AHL and QQ Assays

Fifteen strains, approximately 15% of isolates (21.1% of the Gram-positive strains), were able to inhibit the C6-HSL, C8-HSL and C12-HSL that were exogenously added, indicating their probable QQ activity (Table 2, Figure S3). Among these strains, 46.6% (seven strains) were active against the three AHLs that were analyzed and the remaining 53.4% (eight strains) were positive for two AHLs.

**Figure 1:**
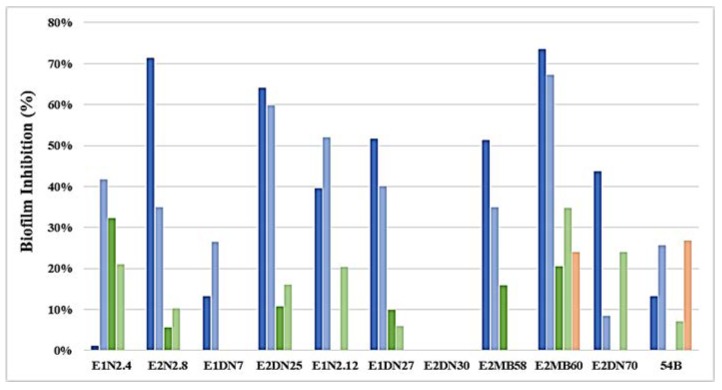
Inhibition of biofilm formation after 24 h incubation in the presence of the eleven strains selected due to their QQ activity. Mixed liquor A (ML_A_), mixed liquor B (ML_B_), and a pure culture of *Pseudomonas aeruginosa* (QS1) were used for biofilm development (meaning de 100% value) and then exposed to the strains E1N2.4, E2N2.8, E1DN7, E2DN25, E1N2.12, E1DN27, E2DN30, E2MB58, E2MB60, E2DN70, and 54B (cell cultures and cultures supernatants). QQ cell cultures in ML_A_ (blue bars), QQ supernatants in ML_A_ (light blue bars), QQ cell cultures in ML_B_ (green bars), QQ supernatants in ML_B_ (light green bars), and QQ supernatants in *P. aeruginosa* cultures (orange bars) were tested.

Based on the previous results, 11 QQ strains belonging to different species and that were able to inactivate long- and medium-chain AHLs were selected to test their effectiveness in reducing biofilm formation using both real wastewater [mixed liquor A (ML_A_) and mixed liquor B (ML_B_)] and monospecific biofilm of a wastewater-relevant strain of *P. aeruginosa*. As shown in Figure 1, the highest inhibition was obtained through the exposition of ML_A_ to the quencher cultures with values that were up to the 60% in the case of the strains E2MB60 (73.38%), E2N2.8 (71.31%), and E2DN25 (64.01%). ML_B_ biofilm reduction showed much lower values than the previous case. Interestingly, better results were assessed with indigenous QQ bacteria than with strains pertaining to the well-known QQ genus *Rhodococcus,* although, regarding the inhibition of monospecific *P. aeruginosa* QS1 (strong producer of long chain AHLs, Table 1) biofilm, only with the use of the strains *R. ruber* 54B and *B. subtilis* E2MB60 (able to degrade only long-chain AHLs, Table 2), were noteworthy results obtained.

**Figure 2:**
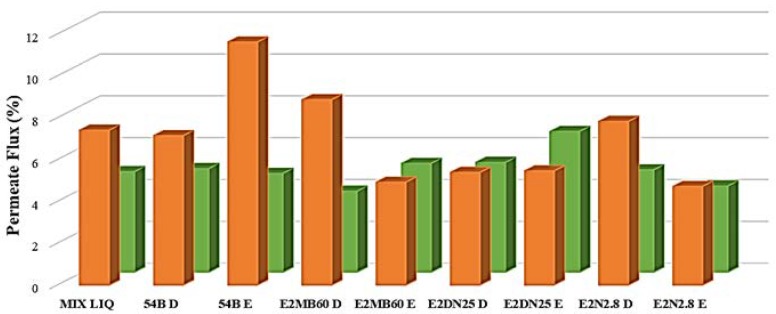
Changes in permeate flux through an ultrafiltration membrane after treatment of water samples with the selected strains. Water samples, from WWTP_A_ (MIX LIQ orange) and WWTP_B_ (MIX LIQ green), were inoculated with the bacterial strains 54B, E2MB60, E2DN25, and E2N2.8 using two methods: direct inoculation of the strains (D) and alginate-entrapped cells (E). Permeate flux was appreciably increased when ML_A_ was treated with strains 54B (E), E2MB60 (D), and E2N2.8 (D).

At this point of our experimental work, the strains *B. subtilis* E2MB60, *B. cereus* E2N2.8, *B. thuringiensis* E2DN25, and *R. ruber* 54B, were selected to study the relationship between QQ activity and biofouling in a UF laboratory plant (see Section 2.8. of *Material and Methods* section). The tested strains were applied directly or were entrapped in alginate spheres. Figure 2 indicates a substantial increase of permeate flux when the mixed-liquor from WWTP-A (ML_A_) was employed in the presence of the entrapped *R. ruber* 54B, suggesting that the immobilization material used might protect this strain from wastewater composition permitting its anti-biofouling ability. Moreover, the results that were obtained with the direct addition of the strain E2MB60 (also to some extent with the E2N2.8), confirmed both, the survival capacity of this specie and its ability as quorum quencher under those environmental conditions. Referring to mixed liquor of WWTP-B (ML_B_), no substantial changes were observed in permeate fluxes when values of control and quenchers presence were compared.

### 3.4. Enzymatic Activities of Selected QQ Strains

#### 3.4.1. Lactonase Activity

**Figure 3:**
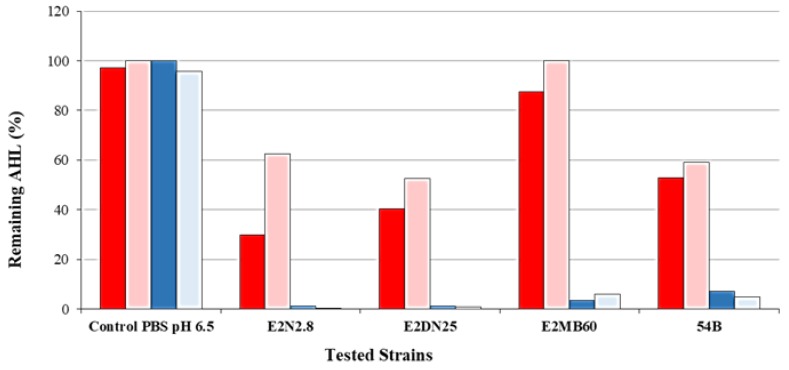
High-pressure liquid chromatography-mass spectrometry (HPLC-MS) analysis of the remaining C6-HSL and C12-HSL after 24 h of incubation with the four selected strains. In order to allow for the recovery of the lactone ring after lactonolysis, culture media containing C6-HSL or C12-HSL were acidified to pH 2.0. C6-HSL (red bars), C6-HSL acidified (light red bars), C12-HSL (blue bars), and C12-HSL acidified (light blue bars). Strains tested were as follows: *B. cereus* E2N2.8, *B. thuringiensis* E2DN25, *B. subtilis* E2MB60, and *R. ruber* 54B.

The capacity of the selected strains to hydrolyze C6- and C12-HSL was confirmed by HPLC-MS. As expected, all four strains were able to reduce the concentration of the C12-HSL, showing more than 90% of QQ activity. This capacity was lower against C6-HSL (Figure 3). The spontaneous lactonization of the AHLs due to high pH was not taken into account since the pH of cultures was lower than seven in all cases. To verify that the QQ activity was due to the action of a lactonase enzyme, cultures containing C6-HSL and C12-HSL were acidified. This allows for the lactone ring to reassemble if it has been previously opened by a lactonase [58,63]. The results showed that the C6-HSL degradation was due to the activity of a lactonase enzyme because the AHL values detected were higher than the non-acidified samples; however, this fact did not occur in the C12-HSL samples, suggesting other enzymatic activity than a lactonase or to be further degraded by a different enzyme after the addition of lactonase, precluding its detection.

#### 3.4.2. Acylase Activity

**Figure 4:**
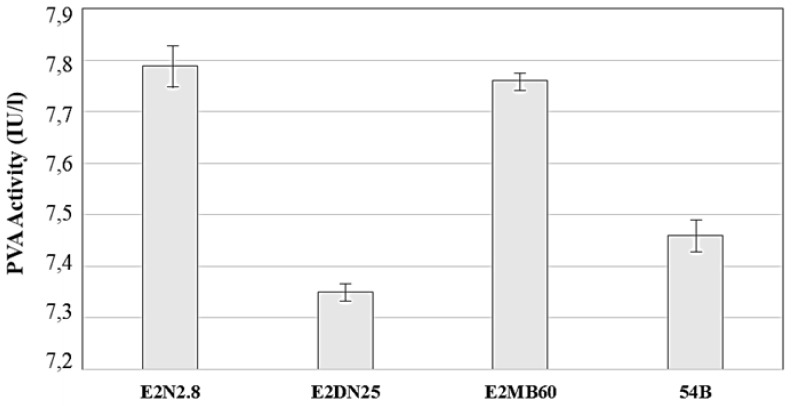
Amount of penicillin V acylase (PVA) activity detected in the supernatants of *B. cereus* E2N2.8, *B. thuringiensis* E2DN25, *B. subtilis* E2MB60, and *R. ruber* 54B. Error bars indicate the standard deviation.

Penicillin acylases bear the ability to cleave penicillin, and, due to wide substrate specificity, also bacterial AHLs [60,64] Penicillin V acylase (PVA) hydrolyzes penicillin V producing 6-aminopenicillanic acid (6-APA) and phenoxyacetic acid [61,65,66], and this mechanism has been used in the present study to determine the presence of acylase activities in the four selected bacterial isolates. As can be seen in Figure 4, all strains that were produced low levels of PVA when grown in LB medium, and, according to the results presented in the same figure, there were no important differences in PVA activity among the selected strains and controls, suggesting that the QQ activity that was ascribed to these strains were probably not due to an acylase activity. It must be considered, for a feasible comparison, that some strains of *Streptomyces lavendulae* [60,61] produce acylase activities in several orders of magnitude higher than the present strains (data not shown).

## 4. Discussion

According to recent publications [19,62,67,68,69,70], QS and QQ processes are very common in natural and anthropic environments, even WWTP. This work shows, in agreement with other studies in WWTP (aerobic granules [71]), the presence in MBR activated sludge of bacterial AHL producers, quenchers, and isolates, with no AHL producing or quenching activity. Among the five AHL-producing isolates (5.1% of the total), three belonged to the *Pseudomonas* genus (two were *P. aeruginosa* species), and the other was *A. hydrophila*, both have been described as key species that are involved in the process of biofilm formation, which causes membrane biofouling in MBR systems [44].

It is remarkable the predominance of AHL quenchers (15) over AHL producers (5). However, this result should be interpreted with caution since it could be a bias derived from the use of culture conditions not resembling the natural environment. A recent study on four different *Escherichia coli* strains showed that the 47% of the variance in expression levels is primarily dependent on the environment (medium-dependent genes) [72]. Even, growing the bacteria in shaken cultures or static conditions was reported to effect on QS AHL profile [70]. Furthermore, the percentage of 15.1% of strains with QQ activity is a high percentage when we compare these results with similar studies in environments, such as soils, where percentages vary among the 2% and the 4.8% [38,39,40], but are comparable to the marine environment, where the percentage was about the 14.4% [55]. The isolation protocol that is employed seems to be decisive and might explain these data, as we decided to use a similar procedure to the one proposed by Romero et al. (2011) [55]. In other studies, AHL-degrading bacteria were isolated following an enrichment procedure based on the utilization of AHLs as sole source of carbon and nitrogen, but the number of isolated quorum quenching bacteria was lower [29,35,73,74].

Among the 15 QQ isolates, 13 of them belonged to the species *B. cereus*, *B. subtilis*, and *B. thuringiensis*, which have been described previously as capable of degrading AHL by the action of a specific gene, aiiA-Autoinducer Inactivator A, encoding a lactonase enzyme that inactivates the AHL by hydrolyzing the core lactone ring [38,39,40,41,42]. Besides, the isolate 54B (*R. ruber*) from the bacterial lab collection was included in our experimental work since the research on *Rhodococcus* genus and specifically the species *R. erythropolis*, *R. ruber*, or *R. qingshengii* have demonstrated their ability to degrade AHLs and to inhibit the formation of biofilm [22,35,36,62,74,75]. In fact, it has been also reported that one strain of *Rhodococcus* (*Rhodococcus* sp. BH4—99% identity with *R. erythropolis* W2) is in effect able to control biofouling in MBR [23,24,26,62], and our strain showed its effectiveness against this problem too. With this aim, we highlight three other adequate candidates for future studies in order to overcome laboratory limitations and be used in pilot-scale QQ-MBR: *B. subtilis* E2MB60, *B. cereus* E2N2.8, and *B. thuringiensis* E2DN25. First, it should be noted that results obtained from biofilm formation tests using those quorum quenchers and the mixed liquors of the wastewater plants were optimistic although a more adequate inoculum of the AHL-degrading strains should be considered. As for the differences that are related to the microbial mixed liquor adherence, the reductions by these species in ML_A_ were higher than in ML_B_. This fact could be explained by the contribution of chemical in ML_B_ (data not shown).

Second, the increase of membrane permeability in an ultrafiltration lab-scale assay while using those strains and the mixed liquor of WWTPs. This assay, in an attempt to indirectly determine whether bacteria quenchers could diminish biofouling, showed an evident reduction of the biofouling that is produced on the membrane with water samples from WWTP_A_, although with water samples from WWTP_B_, there was not almost any flux increase in the presence of QQ bacteria, which could be due to the high concentrations of polyelectrolytes that is used in WWTP_B_, and so on not related to biofouling.

Last, the confirmation of enzymatic QQ activity by the selected isolates using both bacterial biosensors and HPLC-MS techniques. The results showed that the selected strains have the capacity to degrade medium- and long-chain AHLs. Moreover, all of the strains showed that enzymatic activity might be due to a lactonase enzyme because there was a partial recovery of the C6-HSL by posterior acidification, although in the case of C12-HSL, where the recovery of the lactone ring did not occur, the enzymatic activity may be due to an enzyme other than lactonase.

To clarify this aspect, penicillin V acylase (PVA) activity was investigated. According to Mukherji et al. (2014) [64], these enzymes may have the ability to cleave medium chain length AHLs with great efficiency. Although the experimental values obtained demonstrated the presence of PVA, they were low although being comparable to those that were obtained by Rolinson et al. (1961) [76]. According to Torres et al. (1999) [61], the influence of the culture medium plays an important role in the production of this enzyme; therefore, further research will be done in this sense. This is indicated since a recent study [77] shows that PVAs can decrease the biofilm formation of *P. aeruginosa*, however, the low values of activity that were obtained in our study could not be sufficient to inhibit the biofilm formation. In the particular case of *B. subtilis* E2MB60, our good results using this specie could be related to the possible production of subtilosin, a cyclic lantibiotic protein, which has been related to biofilm reduction [78].

## 5. Conclusions

To summarize, this study shows a first approximation for the application of AHL-degrading bacteria to inhibit biofilm formation on membrane bioreactors. Certain strains that were isolated from two MBR wastewater plants treating leachate from municipal solid waste in Spain, showed effectiveness, especially those that were belonging to the genus *Bacillus*. Several studies have been carried out worldwide on this subject however; this is the first time they were performed in Spain. Further pilot-scale QQ-MBR studies will be done based on direct strain addition and/or the encapsulation with alginate in order to verify their utility to solve biofouling problems, and increase the useful lifespan of the membranes, minimizing operational costs.

Further experiments are warranted to gain a better understanding on the practicality of this technology for biofouling control in membrane systems. Currently, additional studies on the QQ activity exerted by the AHL-degrading enzyme acylase from *Actinoplanes utahensis* are under development to inhibit biofilm formation by strains of bacteria (mainly *Pseudomonas aeruginosa*) that were isolated from WWTP. Acylase from *A. utahensis* has shown a 50–70% reduction of biofilm formation by *P. aeruginosa* in both conditions, on solution and attached to borosilicate surfaces.

## Figures and Tables

**Table 1:** Relative estimation of *N*-acyl homoserine lactones (AHL) production assessed by biosensors among Gram negative isolates and the adherence capability of AHL producers.

Bacterial Strains	*C. violaceum CV026*	*A. tumefaciens NT1*	Adherence Capability
*Aeromonas hydrophila* E2MB52	Strong	Moderate	Moderate
*Pseudomonas pseudoalcaligenes* E2MB82	Non-detected	Strong	Strong
*Thauera butanivorans* E2DN83	Non-detected	Moderate	Strong
*Pseudomonas aeruginosa* E2MBUPVDN12	Non-detected	Moderate	Strong
*Pseudomonas aeruginosa* E2MBUPVDN20	Non-detected	Moderate	Strong

**Table 2:** Detection of Quorum Quenching (QQ) activity with the AHL biosensors *Chromobacterium violaceum* CV026 and *Agrobacterium tumefaciens* NT1. QQ-positive strains degraded AHLs (30 mM C6-HSL; 20 mM C8-HSL; 30 mM C12-HSL) after 24 h, inhibiting violacein production and/or X-Gal hydrolysis.

WWTP	Bacterial Strains	C6-HSL	C8-HSL	C12-HSL
WWTPA	*Bacillus cereus* E1DN3	+ ^1^	+	-
WWTPA	*Bacillus subtilis* E1DN6	- ^2^	+	+
WWTPA	*Bacillus* sp. E1DN7	+	+	+
WWTPA	*Gordonia paraffinivorans* E1N2.12	-	+	+
WWTPA	*Bacillus cereus* E1DN27	-	+	+
WWTP B	*Bacillus cereus* E2N2.8	+	+	+
WWTP B	*Bacillus thuringiensis* E2DN25	+	+	+
WWTP B	*Bacillus* sp. E2DN30	-	+	+
WWTP B	*Bacillus cereus* E2DN35	+	+	+
WWTP B	*Bacillus cereus* E2DN36	+	+	+
WWTP B	*Bacillus cereus* E2AL40	+	+	-
WWTP B	*Bacillus cereus* E2AL41	+	+	+
WWTP B	*Bacillus thuringiensis* E2MB58	-	+	+
WWTP B	*Bacillus subtilis* E2MB60	-	+	+
WWTP B	*Bacillus cereus* E2DN70	+	+	+

^1^ (+; degraded), ^2^ (-; non-degraded).

## Supplementary Materials

The following are available online at http://www.mdpi.com/1660-4601/15/5/1019/s1, Figure S1: Scheme of the automated ultrafiltration laboratory plant used in this study, Figure S2: Adherence capabilities of bacterial isolates after 24 h of cultivation determined by the crystal violet assay method, Figure S3: Example of detection of QQ activity with the AHL biosensors *Chromobacterium violaceum* CV026 (a,b) and *Agrobacterium tumefaciens* NT1 (c), Table S1: Bacterial collection with Genbank accession numbers.
