# Supplementary material for: Quorum Sensing versus Quenching Bacterial Isolates Obtained from MBR Plants Treating Leachates from Municipal Solid Waste
Source: Int J Environ Res Public Health. 2018 May 18;15(5):1019. doi: 10.3390/ijerph15051019 (PMC5982058; doi:10.3390/ijerph15051019)
Supplement: Supplementary file 1 [file ijerph-15-01019-s001.pdf]

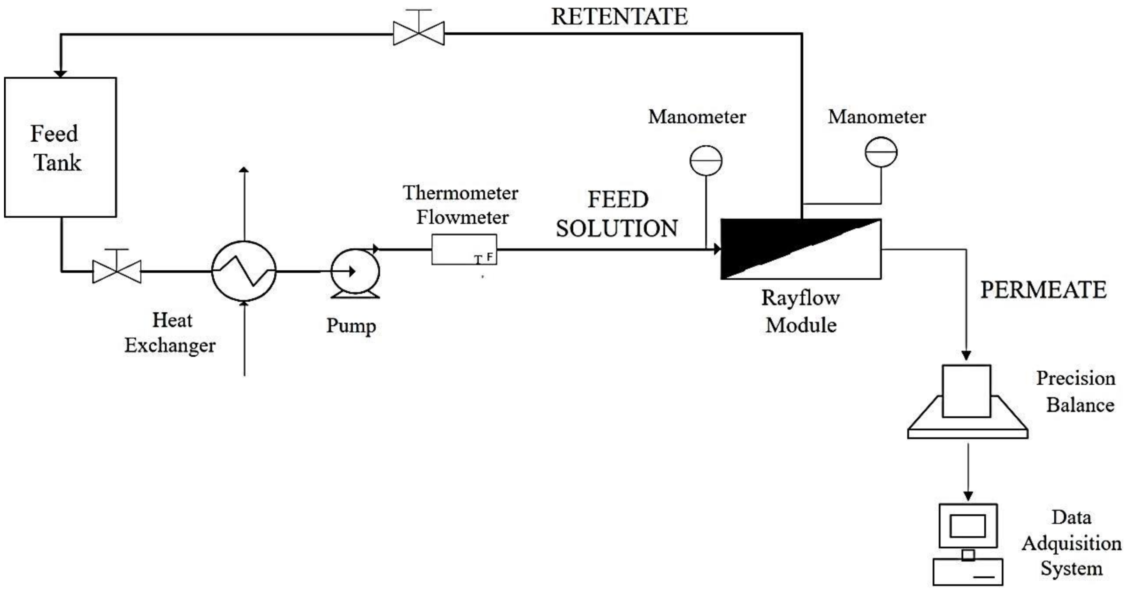

Figure S1. Scheme of the automated ultrafiltration laboratory plant used in this study.

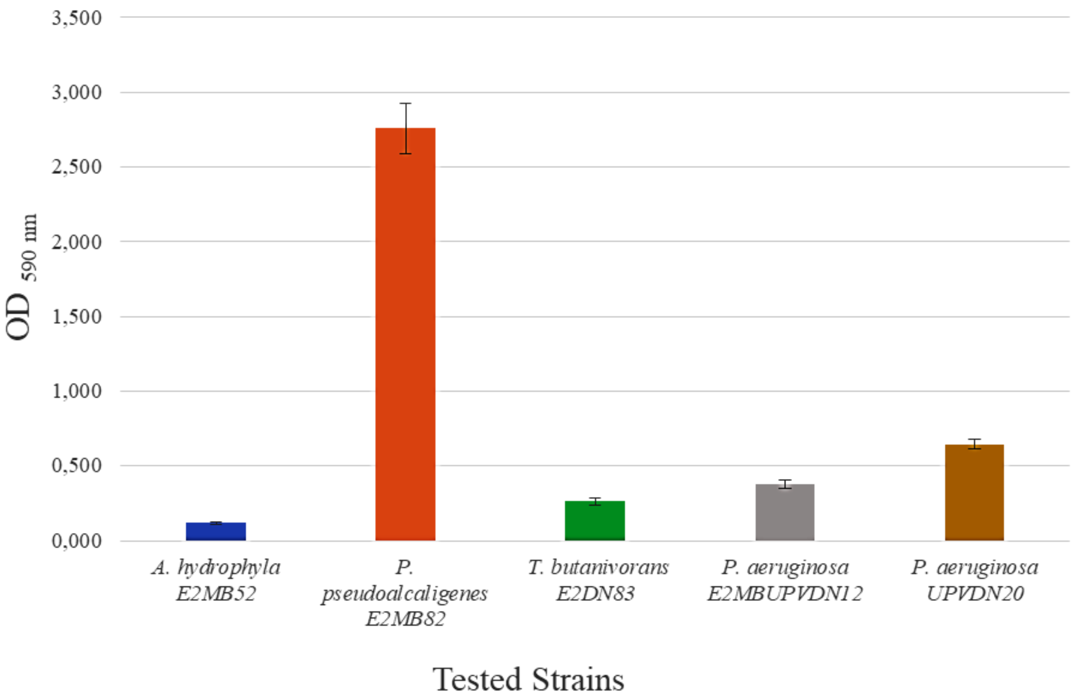

Figure S2. Adherence capabilities of bacterial isolates after 24 h of cultivation determined by the crystal violet assay method. Error bars indicate the standard deviation. OD $_{590nm}$ average control value (LB medium): 0. 060

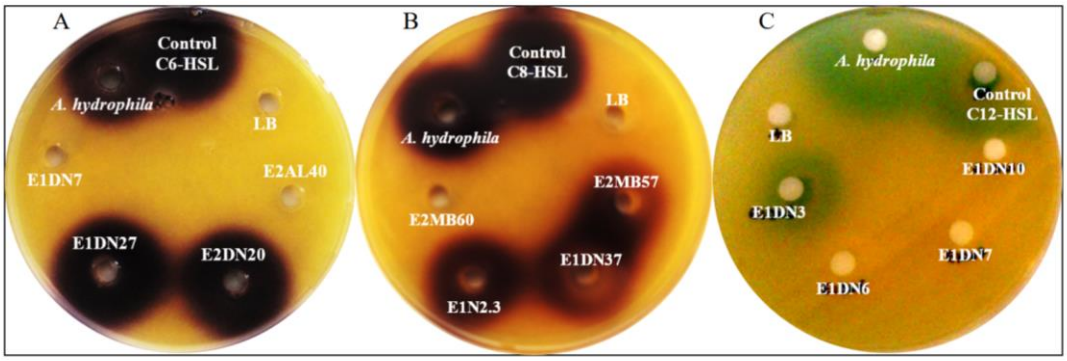

Figure S3. Example of detection of QQ activity with the AHL biosensors *Chromobacterium violaceum* CV026 (a and b) and *Agrobacterium tumefaciens* NT1 (c). QQ-positive strains (E1DN7, E2AL40, E2MB60, E1DN6, E1DN27 and E1DN10) degraded AHLs (30mM C6-HSL; 20mM C8-HSL; 30mM C12-HSL) after 24 h, inhibiting violacein production (a and b) and X-Gal hydrolysis (c). *Aeromonas hydrophila* E2MB52 and AHLs at the same concentrations were used as negative controls (negative QQ activity). LB was also used as control (absence of color production without AHLs).

Table S1. Bacterial collection with Genbank accession numbers.

WWTP	Strain code	Genbank accession number	Identification (species)	Similarity (%)
WWTP1	E1DN3	KU897070	*Bacillus cereus*	99%
WWTP1	E1DN6	KU897071	*Bacillus subtilis*	99%
WWTP1	E1DN7	KU897072	*Bacillus megaterium*	100%
WWTP1	E1N2.12	KU897073	*Gordonia paraffinivorans*	99%
WWTP1	E1DN27	KU897074	*Bacillus cereus*	99%
WWTP2	E2N2.8	KU897075	*Bacillus cereus*	99%
WWTP2	E2DN25	KU897076	*Bacillus thuringiensis*	99%
WWTP2	E2DN30	KU897077	*Bacillus subtilis*	99%
WWTP2	E2DN35	KU897078	*Bacillus cereus*	99%
WWTP2	E2DN36	KU897079	*Bacillus cereus*	99%
WWTP2	E2AL40	KU897080	*Bacillus cereus*	99%
WWTP2	E2AL41	KU897081	*Bacillus cereus*	99%
WWTP2	E2MB58	KU897082	*Bacillus thuringiensis*	99%
WWTP2	E2MB60	KU897083	*Bacillus subtilis*	100%
WWTP2	E2DN70	KU897084	*Bacillus cereus*	99%
WWTP2	E2MB82	KU897085	*Pseudomonas pseudoalcaligenes*	100%
WWTP2	E2DN83	KU897086	*Thauera butanivorans*	99%
WWTP2	E2MBUPVDN12	KU897087	*Pseudomonas aeruginosa*	99%
WWTP2	E2MBUPVDN20	KU897088	*Pseudomonas aeruginosa*	99%
WWTP2	E2MB52	KU897089	*Aeromonas hydrophila*	99%
WWTP La Rábida	54B	KU597170	*Rhodococcus ruber*	100%
